# Modelling the gene expression and the DNA-binding in the 3T3-L1 differentiating adipocytes

**DOI:** 10.1080/21623945.2019.1697563

**Published:** 2019-12-06

**Authors:** Mahmoud Ahmed, Deok Ryong Kim

**Affiliations:** Department of Biochemistry and Convergence Medical Sciences and Institute of Health Sciences, Gyeongsang National University School of Medicine, Jinju, Republic of Korea

**Keywords:** Gene-expression, RNA-Seq, DNA-binding, ChIP-Seq, adipocyte-differentiation

## Abstract

The 3T3-L1 pre-adipocyte cell line is widely used to study the fat cell differentiation *in vitro*. Researchers also use this cell model to study obesity and insulin resistance. We surveyed the literature, the gene expression omnibus and the sequence read archive for RNA-Seq and ChIP-Seq datasets of MDI-induced 3T3-L1 differentiating cells sampled at one or more time points. The metadata of the relevant datasets were manually curated using unified language across the original studies. The raw reads were collected and pre-processed using a reproducible state-of-the-art pipeline. The final datasets are presented as reads count in genes for the RNA-Seq and reads count in peaks for the ChIP-Seq dataset. The curated datasets are available as two Bioconductor experimental data packages curatedAdipoRNA and curatedAdipoChIP. In addition, the packages document the source code of the data collection and the pre-processing pipelines. Here, we provide a descriptive analysis of the datasets with context and technical validation.

## Introduction

The 3T3-L1 cell line is used as a cell model for studying the fat cell differentiation [1]. This adipocyte differentiation model has many applications in obesity and insulin-resistance research such as lipid synthesis, white vs brown adipose tissue development, insulin-sensitizing drug action [2–4]. The most commonly investigated aspect of the molecular biology of this cell line is the gene expression and chromatin binding at the different stages of differentiation. The development of the phenotype is achieved through certain transcription factors which drive a well-defined transcriptional program [5]. High-throughput sequencing technologies are used to model the connection between gene expression and chromatin in the transcriptional regulation. The availability of sufficient sample size and good quality datasets is a necessity for successful modelling.

The increasing amount of available high-throughput sequencing data necessitates the development of standards for sharing and integrating data. The creators of the primary data are often required to adhere to the standards of the repository where they report and share the data. The development of across repositories metadata standards, ontologies and controlled vocabularies has been attempted to help researchers to share the data they generate and use the data generated by others [6]. Despite the fact that these attempts are general in purpose and intended to work across different data types, we found them to be useful in curating the metadata of the specific adipocyte model [7]. In particular, we used standard model metadata such as induction media, culturing time and the antibodies to encode the metadata necessary for understanding the experimental design across different studies. Confounder metadata such as library type and machine model has been also recorded to facilitate the analysis of the data.

We surveyed the literature, the gene expression omnibus (GEO) and the sequence read archive (SRA) for RNA-Seq and ChIP-Seq datasets of differentiating 3T3-L1 cells sampled at one or more time points [8,9]. The metadata of the relevant datasets was manually curated using unified language across the different studies. The data were processed using an updated reference genome and annotation. The final product was packaged in a versatile object format that allows for any number of downstream analysis. The curation of a large number of samples from similarly designed experiments can be useful [10]. In addition, these datasets were processed in standard pipelines to allow combining, comparing and integrating data from different studies or sources. In other words, this work increases the utility of the datasets by providing data ready for exploring and testing hypotheses.

In this article, we present the methods that were used to collect and process the raw data and provide a technical validation of the final product. We start by describing the cell line model and the induction protocol for adipocyte differentiation. Then, we state the search strategy and the inclusion criteria of the studies. Next, we present the steps and the tools for obtaining and processing the raw data. Finally, we provide links to the software environment and the code for reproducing the full process. Using a subset of the data, we perform several technical validation analyses. First, we check the separation of the samples by phenotype in multidimensional scaling (MDS) and the sample replication using similarity measures. Second, we describe the expression and binding patters of adipocyte markers and enrichment of gene sets which are expected true biology for this model. Moreover, we compared several aspects of the model to human primary adipocytes. Together, the descriptive analysis provides an assessment for the validity of the model and the appropriateness of the curation process.

## Materials and methods

### 3T3-L1 differentiation protocol

3T3-L1 is a mouse pre-adipocyte cell line which can be induced to differentiate into mature adipocytes when it is treated with a chemical cocktail. The most commonly used variant of these chemical cocktails contains 1-Methyl-3-isobutylxanthine, Dexamethasone and Insulin (MDI). The treatment usually starts by inducing fully confluent 3T3-L1 cell culture (pre-adipocyte) which begins to differentiate shortly after by accumulating lipid into lipid droplet structures within days [1]. Another variant of these differentiation media includes the addition of rosiglitazone, a peroxisome proliferator-activated receptor gamma (PPARG) agonist [11]. In this dataset, we selected the studies that used the MDI differentiation protocol with minimal variations in dose and method. The differentiation course is usually divided into several stages depending on the expression of certain markers and the amount of accumulated lipid; the day 2–4 marks the early stage; up to day 7 is an intermediate stage and up to 14 d is late-differentiation stage.

### Data collection and acquisition

We surveyed GEO and SRA repositories for high-throughput sequencing data of MDI-induced 3T3-L1 pre-adipocyte samples at different time points. The data were obtained from GEO or SRA in the form of raw reads (fastq). In total, 98 RNA-Seq and 187 ChIP-Seq samples (transcription factor, co-factor and histone modification markers; referred to as factors) were included. Samples with multiple runs or paired-end runs were obtained separately and combined in later steps of the pipeline. Raw reads were downloaded from the SRA ftp server using Wget. FASTQC was used to assess the quality of the raw reads [12]. We did not remove the samples with low quality at this stage but rather added the quality information to the final product in the metadata table in the form of qc_read objects.

### Data records

Table 1 and 2 list the RNA-Seq and ChIP-Seq datasets included in this curation, respectively. For each dataset, we recorded The GEO/SRA ID, the number of included sample (N), the time points in hours from the point of MDI induction and the stage of differentiation (0, non-induced; 1, early; 2, intermediate; 3, late-differentiation). Each dataset was connected to a published study of which we recorded the PubMed ID and a reference.

**Table 1.** Gene expression RNA-Seq datasets. GEO, gene expression omnibus; PMID, PubMed ID; N, number of samples; Stage, stage of differentiation (0, non-induced, 1, early; 2, intermediate; 3, late-differentiation); Ref., reference; NA, missing.

GEO ID	PMID	(N)	Time (hr)	Stage	Ref.
GSE100056	29,138,456	4	−48/24	0/1	[13]
GSE104508	29,091,029	3	192	3	[14]
GSE35724	24,095,730	3	192	3	[15]
GSE50612	25,614,607	8	−48/0/10/144	0/1/3	[16]
GSE50934	24,912,735	6	0/168	0/3	[17]
GSE53244	25,412,662	5	−48/0/48/120/240	0/1/3	[18]
GSE57415	24,857,666	4	0/4	0/1	[19]
GSE60745	26,220,403	12	0/24/48	0/1	[20]
GSE64757	25,596,527	6	168	3	[21]
GSE75639	27,923,061	6	−96/-48/0/6/48/168	0/1/3	[22]
GSE84410	27,899,593	6	0/4/48/28	0/1	[23]
GSE87113	27,777,310	6	0/1/2/4/48/168	0/1/3	[24]
GSE89621	28,009,298	3	240	3	[25]
GSE95029	29,317,436	10	0/48/96/144/192	0/1/2/3	[26]
GSE95533	28,475,875	10	4/0/24/48/168	1/0/3	[27]
GSE96764	29,748,257	6	0/2/4	0/1/2	[28]

**Table 2.** DNA-binding ChIP-Seq datasets. SRA, sequence read archive; PMID, PubMed ID; N, number of samples; Stage, stage of differentiation (0, non-induced, 1, early; 2, intermediate; 3, late-differentiation); Factor, protein antibody; Ref., reference; NA, missing.

SRA ID	PMID	(N)	Time (hr)	Stage	Factor	Ref.
SRP000630	18,981,474	18	0/24/48/72/96/144	0/1/2/3	PPARG/RXRG/POLR2A	[29]
SRP002283	20,442,865	1	NA	3	E2F4	[30]
SRP002337	20,887,899	15	−48/0/48/168	0/1/3	H3K4me3/H3K27me3/H3K36me3/H3K4me2/H3K4me1/H3K27ac/PPARG	[69]
SRP002507	20,478,996	2	0/6	0/1	CEBPB	[31]
SRP006001	21,427,703	13	0/2/4/48/144	0/1/3	CEBPB/CEBPD/NR3C1/STAT5A/RXRG/PPARG/POLR2A	[32]
SRP008061	21,914,845	1	24	1	TCF7	[33]
SRP009613	24,315,104	6	NA	0	PPARG/JUN/CREB1/PSMB1/Ubiquitin	[34]
SRP016054	23,178,591	4	NA/168	0/3	H3K4me3/H3K27me3/H3K9me2	[35]
SRP028367	23,885,096	7	168	3	PPARG/MED1/CEBPA/POLR2A/CREB1	[36]
SRP029985	24,912,735	3	0/168	0/3	KDM1A/NRF1	[17]
SRP041129	24,788,520	10	NA	3	MED1/CREB1/EP300/NCOR1/CEBPA/CEBPB/ATF2/JUND/FOSL2	[37]
SRP041249	24,857,652	19	4	1	ATF2/ATF7/JUN/FOSL2/KLF4/KLF5/PBX1/STAT1/VDR/RXRG/MED1/EP300/SMARCA4/H3K27ac/H3K4me1/H3K4me2	[38]
SRP042079	24,953,653	2	0	0	GPS2	[39]
SRP043216	25,503,565	8	NA	0	H3K27ac/H3K27me3/H3K36me3/H3K4me1/H3K4me3/H3K79me2/H3K79me3/H4K20me1	[40]
SRP064188	26,590,716	11	0/144	0/3	H3K27me3/H3K9me3/SETDB1/MBD1/POLR2A	[41]
SRP065028	28,398,509	1	168	3	KMT2B	[42]
SRP078506	27,899,593	14	0/4/48	0/1	H3K4me3/KDM5A/KDM5C	[23]
SRP080809	28,107,648	2	NA	0	CEBPB	[43]
SRP100871	28,475,875	52	4/0/48/96/168	1/0/2/3	CTCF/H3K27ac/H3K4me1/H3K4me2/HDAC2/HDAC3/MED1/NCOR1/EP300/SMC1A	[27]

### Data pre-processing and processing

#### Gene expression data processing pipeline

**Figure 1.**
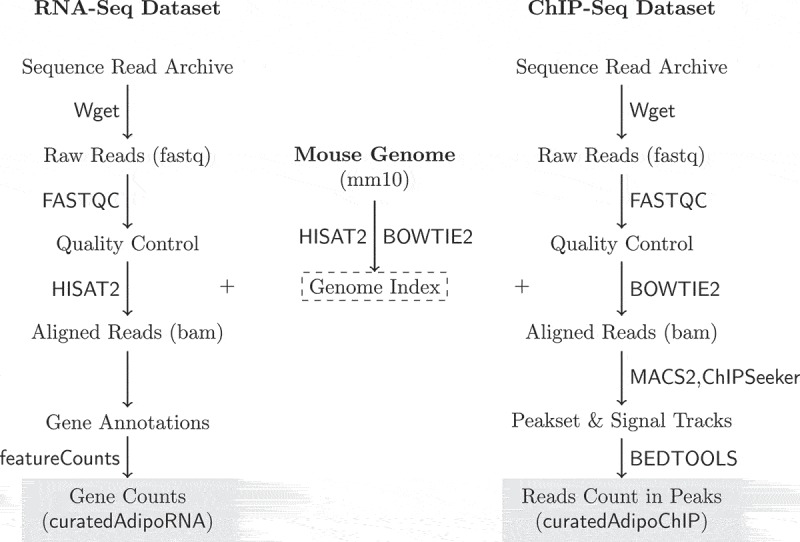
RNA-Seq and ChIP-Seq data processing pipelines. Raw reads were obtained from SRA using Wget. Reads quality was assessed using FASTQC. The mouse genome sequence and annotation were downloaded from UCSC. The genome indices were generated and used to align the RNA and ChIP-Seq reads using HISAT2 and BOWTIE2, respectively. The aligned RNA-Seq reads were used to count reads in genes using featureCounts. The aligned ChIP-Seq reads were used to call and annotate peaks using MACS2 and ChIPSeeker. Reads in peaks were counted using BEDTOOLS.

For RNA-Seq, the raw reads were aligned to UCSC mm10 mouse genome using HISAT2 [44]. FeatureCounts was used to count the aligned reads (bam) in known genes [45]. The reads count in genes were presented as a count matrix with genes in rows and samples in columns. Together, the metadata of the samples, the gene annotations and the count matrix were packaged in a SummarizedExperiment object and deposited as a Bioconductor experimental data package (curatedAdipoRNA) [46]. Figure 1 (*left*) depicts the steps of processing the RNA-Seq data.

#### DNA-binding data processing pipeline

For ChIP-Seq, the raw reads were aligned to the same mm10 genome using BOWTIE2, peaks and signal tracks were built from the aligned reads (bam) using MACS2 [47,48]. The reads count in a peakset of replicated peaks across samples was acquired and arranged in a matrix with peaks in rows and samples in columns using BEDTOOLS [49]. The peakset was annotated and peaks were assigned to the nearest gene using ChIPseeker [50]. Genomic annotations and gene coordinates were accessed through TxDb.Mmusculus.UCSC.mm10.knownGene [51]. As described above, the metadata and the data were arranged in a SummarizedExperiment object and deposited as a Bioconductor experimental data package (curatedAdipoRNA) [46]. Figure 1 (*right*) depicts the steps of processing the ChIP-Seq data.

### Method of technical validation

The technical validation analysis presented in this manuscript was based on the reads count in genes or peaks from the RNA-Seq (n = 98) and ChIP-Seq (n = 22, subset) samples of the curated datasets. The counts were transformed using the variance stabilization transformation (VST) to adjust the distribution of counts to be comparable across samples. MDS was applied using cmdscale (base R) [52]. The differential expression analysis was applied using DESeq2 [53]. The gene ontology (GO) terms annotation was obtained from org.Mm.eg.db and tested for enrichment using goseq [54,55]. The R packages tidyverse, xtable and ComplexHeatmap were used to transform, reshape and visualize the data [56–58]. The analysis was conducted in an R environment and using Bioconductor packages [59,60].

The signal tracks from histone modification ChIP-Seq samples (n = 9) were built from the aligned reads using MACS2 [48]. The scores over 10 bp windows in the promoter regions (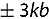) around the transcription start sites of the genes of interest were extracted, normalized and visualized using EnrichedHeatmap [61]. Three datasets of human primary adipocytes were used to compare the gene expression and DNA-binding in 3T3-L1 model to primary cells. Isolates from the subcutaneous fat of healthy subjects (n = 24) were induced for differentiation using MDI for 10 d and profiled for gene expression by microarrays (GSE98680) [62]. Human mesenchymal stem cells (hMSC) and human multipotent adipose-derived (hMAD) cells were induced by the same medium for 6 h or 19 d and used in ChIP-Seq for CEBPB (GSE68864) or PPARG (GSE59703), respectively [63,64]. The processed data were obtained from GEO using GEOquery [65].

### Software environment and code availability

We packaged the software environment where the code was executed as docker images (https://hub.docker.com/r/bcmslab/adiporeg). The scripts used to collect, process and package the datasets are available on GitHub under GPL-3 licence (https://github.com/MahShaaban/curatedAdipoRNA and https://github.com/MahShaaban/curatedAdipoChIP). The code for generating the technical validation figures and the metadata tables in this manuscript is available on GitHub (https://github.com/BCMSLab/curated_adipo_describtor).

## Results and discussion

### The stage of differentiation explains the variance among the adipocyte samples

**Figure 2.**
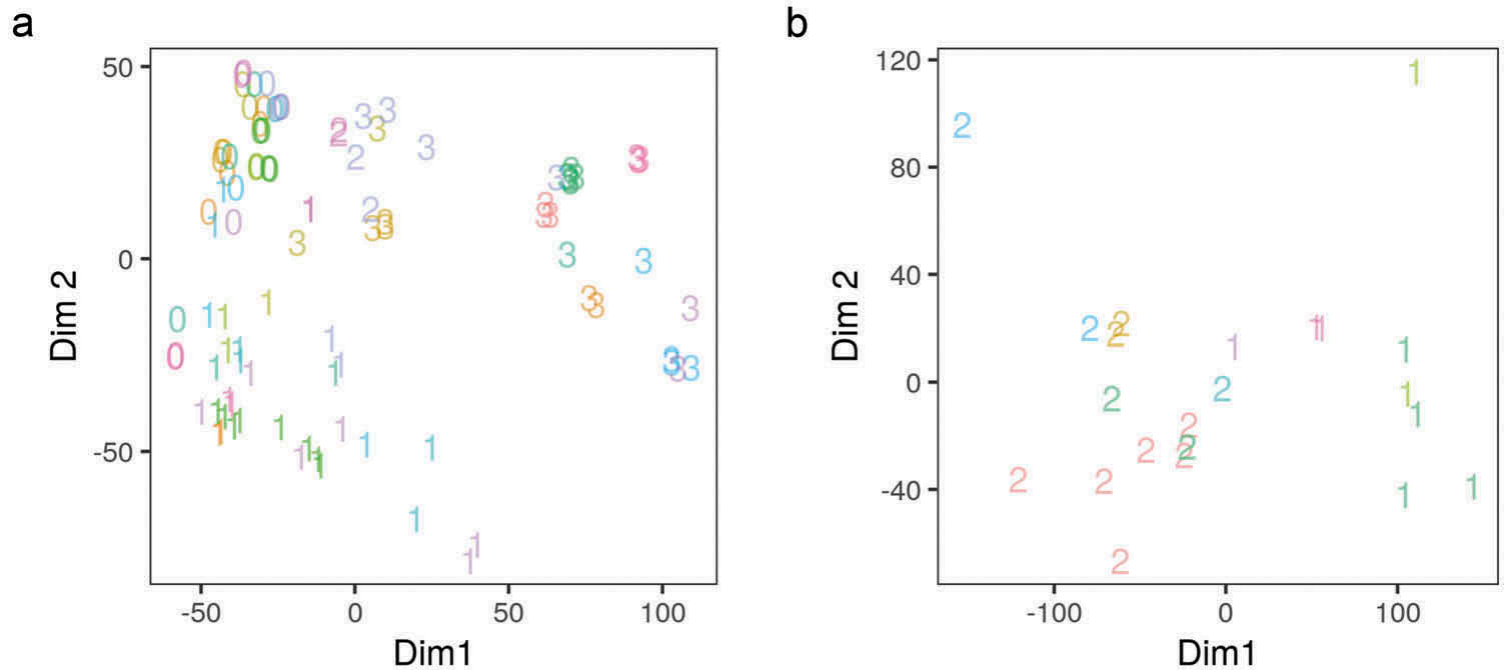
Multidimensional scaling analysis of the gene expression and adipogenic transcription factor binding in differentiating adipocytes. (a) Reads count in genes from RNA-Seq samples (n = 98) were transformed using variance stabilization transformation (VST) and used as an input to multidimensional scaling (MDS). The first two dimensions are shown. Numbers represent the differentiation stage of the samples (0, non-induced; 1, early; 2, intermediate; 3, late-differentiation). (b) Reads count in peaks of a subset of ChIP-Seq samples (n = 22) were transformed using VST and used as an input in MDS. The first two dimensions are shown. Numbers represent the antibody used in each sample (1, CEBPB; 2, PPARG). Colours represent the origin study of the sample.

To test whether or not the final-processed datasets represent the distinct phenotypes they are supposed to, we applied MDS analysis on the full RNA-Seq reads count in all genes and a subset of the ChIP-Seq reads count in peaks of CCAAT enhancer binding protein beta (CEBPB) and PPARG targets. The counts were first transformed using VST. The stage of differentiation of the samples (0–3) was used to represent the phenotype and it showed an appropriate separation along the first two dimensions of the MDS (Figure 2(a)). The stage of differentiation but not the dataset study origin variable explained a significant amount of the variance in gene expression. Similarly, the DNA-binding patterns were explained by the factor/antibody used in each sample (Figure 2(b)).

### Adipocytes at the same stage and their replicates are similar to each other and dissimilar to other stages

**Figure 3.**
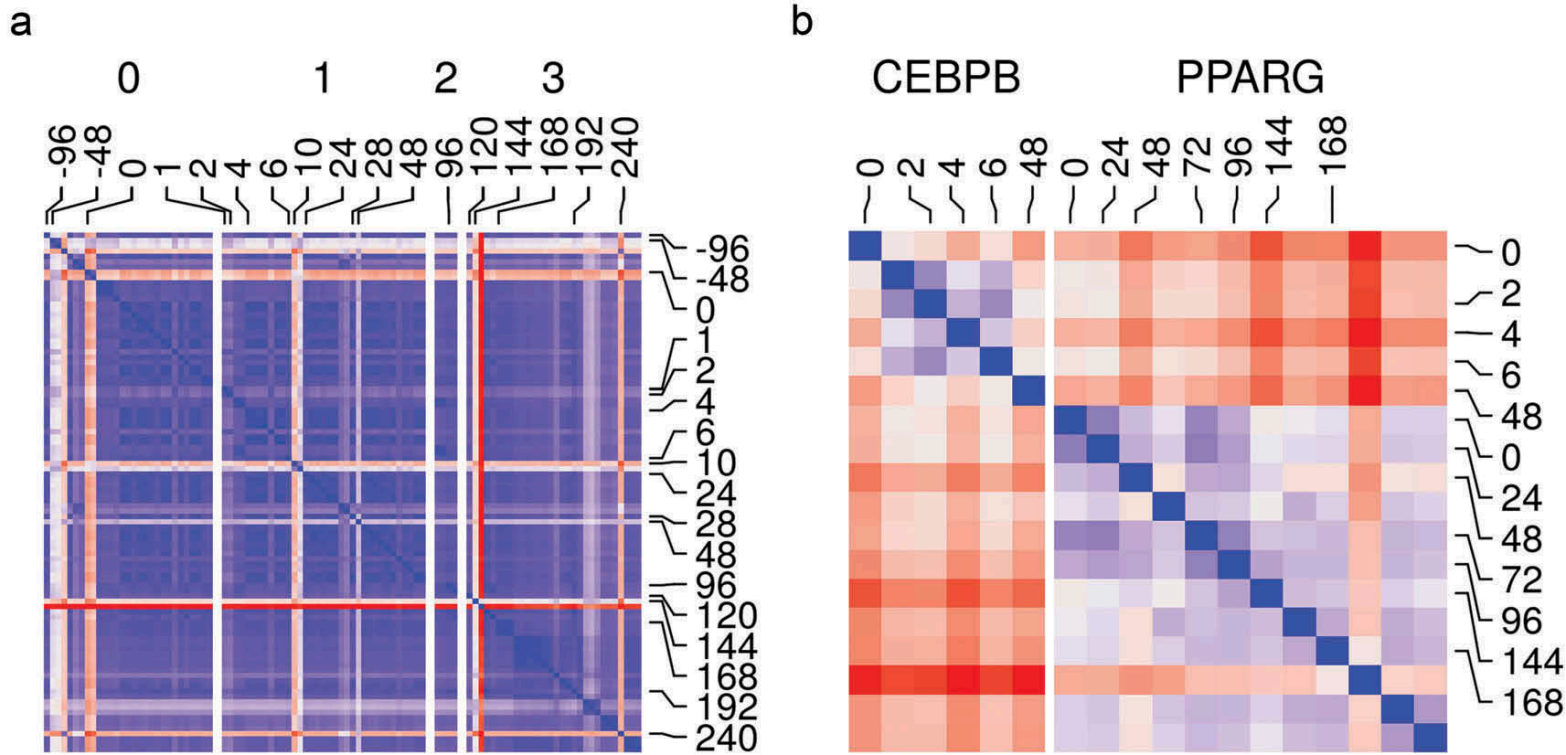
Samples and replicates similarity. (a) Reads count in genes from RNA-Seq samples (n = 98) were transformed using variance stabilization transformation (VST) and used to calculate *Euclidean* distances between all pairs of samples (*blue*, low; *red*, high). Samples are labelled by their time (hours) and the differentiation stage (0, non-induced; 1, early; 2, intermediate; 3, late-differentiation). (b) Reads count in peaks from ChIP-Seq samples (n = 18) were transformed using VST and used to calculate *Euclidean* distances between all pairs of samples (*blue*, low; *red*, high). Samples are labelled by their time (hours) and the antibody used in the sample (CEBPB or PPARG).

We tested the relationship among different samples and replicates. We used the counts from RNA-Seq and ChIP-Seq samples to calculate the *Euclidean* distances among them as a measure of dissimilarity. With the exception of a few samples, most RNA-Seq samples had low dissimilarity between replicates and close-by phenotype (time point/stage of differentiation) (Figure 3(a)). This suggests adequate data filtering and pre-processing. In addition, the gene expression reflects the distinct genotype of the adipocyte at a different stage of maturation. The ChIP-Seq samples from the same ChIP antibody were also similar and they had a low dissimilarity by phenotype within each group (Figure 3(b)). Therefore, the binding pattern from each sample can be replicated for a given factor and distinguished enough from that of other factors.

### Adipocytes exhibit appropriate gene expression and binding patterns of adipogenic and lipogenic markers

**Figure 4.**
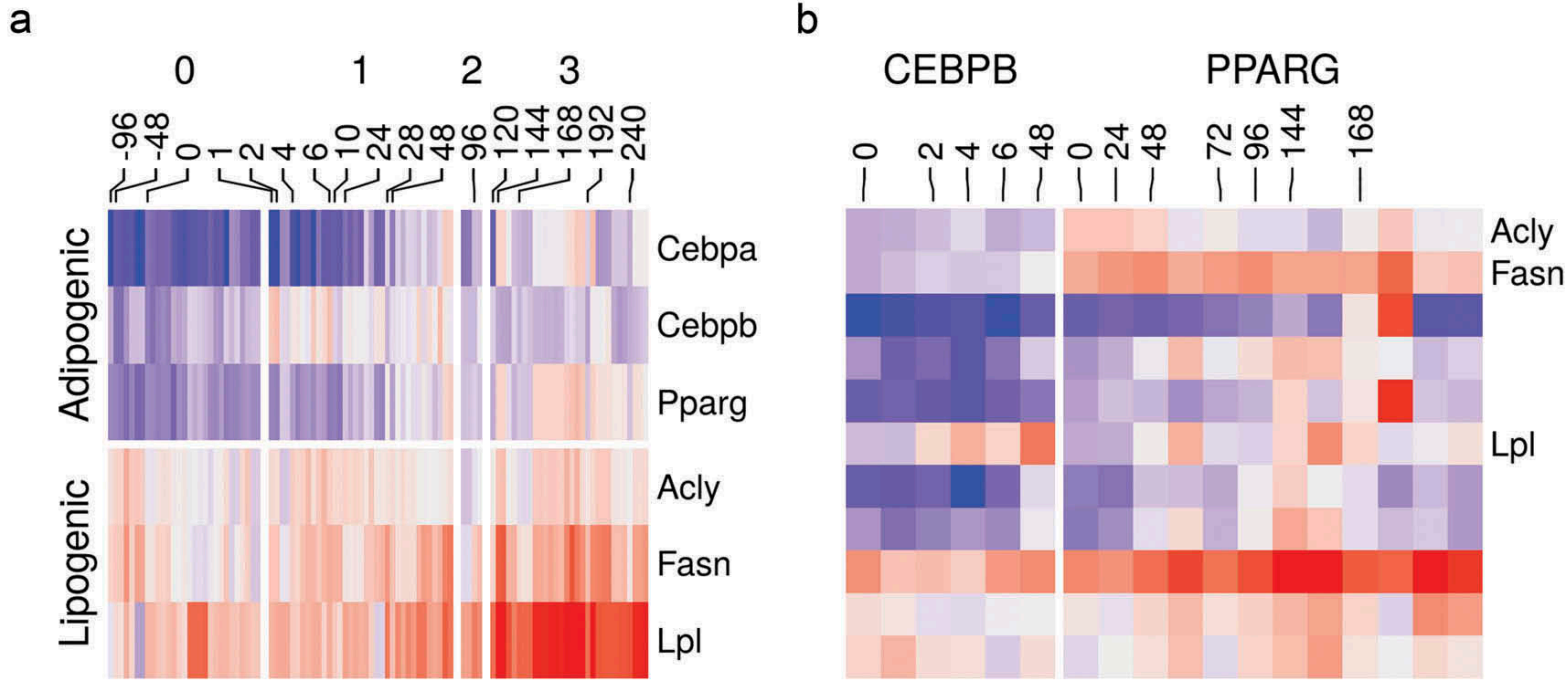
Gene expression and binding patterns of adipogenic transcription factor and lipogenic genes. (a) Reads count in *adipogenic* (*Cebpa, Cebpb* and *Pparg*) and *lipogenic* (*Acyl, Fasn* and *Lpl*) genes from RNA-Seq samples (n = 98) were transformed using variance stabilization transformation (VST), scaled and shown as colour values (*blue*, low; *red*, high). Samples are labelled by their time (hours) and the differentiation stage (0, non-induced; 1, early; 2, intermediate; 3, late-differentiation). (b) Reads count in peaks of lipogenic genes (same as above) from ChIP-Seq samples (n = 22) were transformed using VST, scaled and shown as colour (*blue*, low; *red*, high). Samples are labelled by their time (hours) and the antibody used in the sample (CEBPB or PPARG).

The adipocyte differentiation is a well-studied process. In response to the induction stimulus, certain adipogenic factors are turned on. They direct a well-defined transcriptional program which transforms the cell into the mature adipocyte characterized by the accumulation of lipids in the lipid droplets [5,66]. Therefore, we could use this information to confirm that the curated datasets reflect meaningful biology as expected from high-quality experiments. The expression of essential adipogenic transcription factor genes *Cebpb* and *Pparg* was turned on at the early (stage 1) and intermediate-late (stage 2–3) differentiation stages, respectively (Figure 4(a)). The expression of important lipogenic genes such as *Acly, Fasn* and *Lpl* which are essential for lipid synthesis and accumulation was progressively increased as the cells transitioned to the mature adipocytes (Figure 4(a)). The same was also reflected by the increased binding of CEBPB and PPARG in peaks belonging to the lipogenic genes (Figure 4(b)).

### Adipose, lipid and insulin-related gene sets are enriched in differentiated adipocytes compared to pre-adipocytes

**Figure 5.**
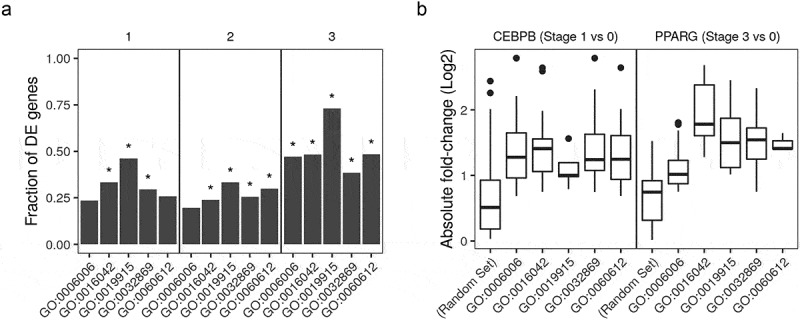
Gene ontology enrichment analysis of differentially expressed genes and differentially bound peaks. (a) Genes from RNA-Seq samples (n = 98) were tested for differential expression between stages (1, early; 2, intermediate; 3, late-differentiation) and 0, non-induced stage using the reads count. The deferentially expressed (DE) genes were used to perform gene set enrichment analysis. The fraction in each comparison of DE genes in the gene ontology (GO) terms: *adipose tissue development* (GO:0060612); *lipid catabolic process* (GO:0016042); *lipid storage* (GO:0019915); *glucose metabolic process* (GO:0006006); and *cellular response to insulin stimulus* (GO:0032869) are shown as bars. (*) indicates p-values 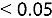. (b) Peaks in ChIP-Seq samples (n = 22) were tested for differential peak binding between stage (1, early-differentiated, for CEBPB; 3, late-differentiated, for PPARG) and 0 non-induced stage using the reads count in peaks. The absolute fold-changes of significantly expressed peaks in genes from the three GO terms (same as above) and a random gene set (n = 50) are shown as box plots (25%, 50% and 75% percentiles).

We used the differentially expressed (DE) genes and differentially bound peaks to perform gene set enrichment analysis of key biological processes-gene ontology (GO) terms – that are most expected to be actively regulated during the differentiation course. The terms *adipose tissue development* (GO:0060612); *lipid catabolic process* (GO:0016042); *lipid storage* (GO:0019915); *glucose metabolic process* (GO:0006006); and *cellular response to insulin stimulus* (GO:0032869) were enriched in most comparisons. The fraction of DE gene members of each term between induced (stage 1–3) and non-induced (stage 0) samples was significantly higher than what is expected by chance alone (Figure 5(a)). The same pattern was also observed in the binding pattern on the gene members of the same terms that are bound to CEBPB or PPARG in induced (stage 1 or 3, respectively) and the non-induced (0 stage) samples. These peaks had absolute (up or down) fold-changes more than the random gene set (Figure 5(b)).

### The regulation of adipose development genes is associated with expected histone modifications

**Figure 6.**
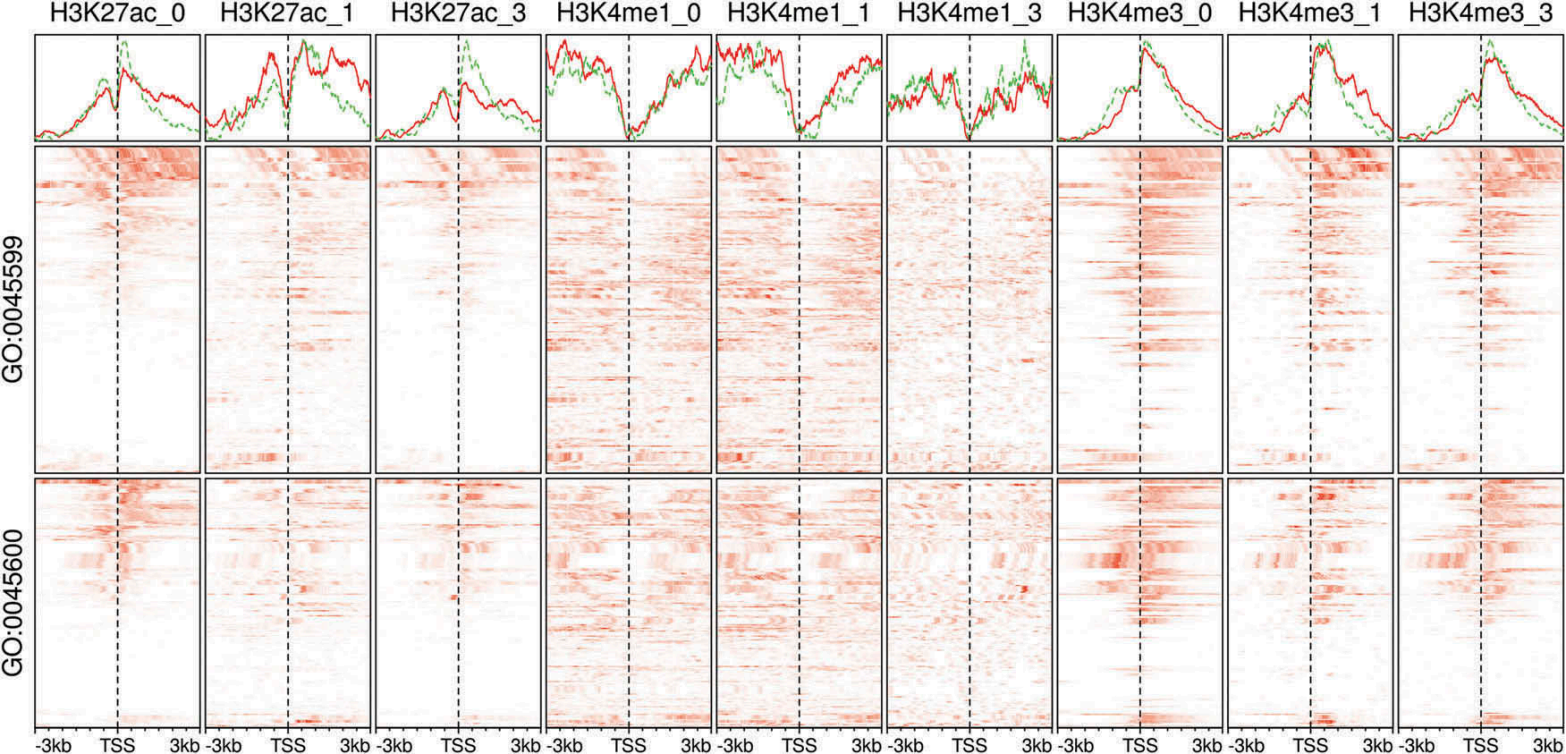
Histone modifications at the promoter region of fat cell differentiation regulators in adipocytes. Signal tracks from ChIP-Seq samples (n = 9) of histone markers were extracted from regions coding for the members of two gene ontology (GO) terms. The GO terms are *negative regulation of fat cell differentiation* (GO:0045599; n = 63) and *positive regulation of fat cell differentiation* (GO:0045600; n = 62). Scores at 10 bp windows over genomic regions of 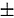
3kb around the transcript start site (TSS) are shown as heatmaps (red). The average scores over the same genomic regions are shown as separate line for GO:0045599, green and GO:0045600, red. Samples are labelled by the histone marker ChIP antibody and the differentiation stage (0, non-induced; 1, early; 3, late-differentiation).

Histone modifications play an important role in the adipocyte differentiation [67]. They assume roles in the induction and/or the repression of adipogenic genes by working either individually or in combinations [68]. Moreover, histone markers are usually found in association with transcription factors at the enhancers and promoter regions [69]. Here, we showed as an example the dynamic changes in the modification patterns of H3K27ac, H3K4me1 and H3K4me3 at the promoter regions of the members of two important gene ontology terms *negative regulation of fat cell differentiation* (GO:0045599) and *positive regulation of fat cell differentiation* (GO:0045600). The modifications varied across two variables; the stage of differentiation and the functional category of genes (Figure 6). The changes suggest the dynamic modification of genes in key pathways where the induced and repressed expression had different signatures; however, an exhaustive study of these signatures is beyond the purpose of the current validation.

### The 3T3-L1 model reflects essential aspects of the adipocyte biology

**Figure 7.**
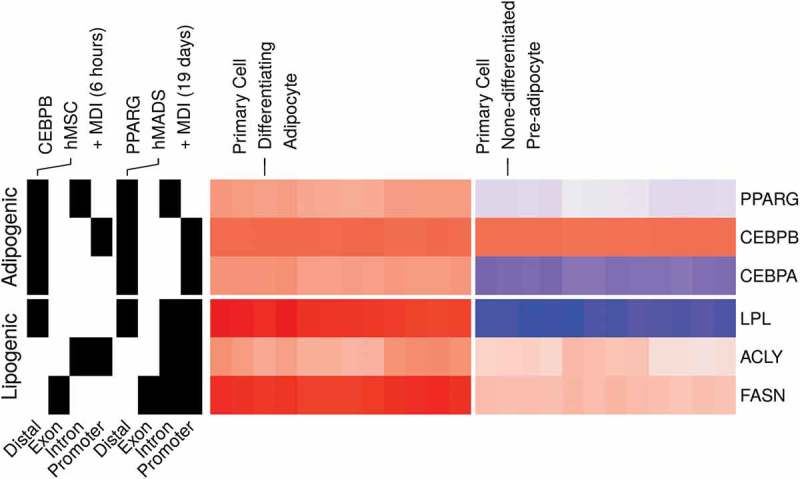
Gene expression and binding patterns of adipogenic transcription factors and lipogenic genes in human primary adipocytes. Probe intensities from microarrays samples (n = 24) from primary pre-adipocytes and differentiating adipocytes were used to estimate the expression (low, blue; high, red) of selected genes (GSE98680). Samples were prepared from primary cells isolated from the subcutaneous fat of healthy human subjects. The isolated cells were induced for differentiation using MDI medium for 10 d. The peaks from ChIP-Seq samples from primary adipocytes were used to represent the binding (present, black; not, white) of adipogenic transcription factors on selected genes. CEBPB and PPARG ChIP antibodies were used in human mesenchymal stem cells (hMSC) (GSE68864) or human multipotent adipose-derived (hMAD) (GSE59703) cells 6 h or 19 d after MDI-induction.

The 3T3-L1 cell line provides a model for studying the development of the fat cells and the behaviour of the mature adipocytes [70]. Although differences are expected to arise between the model and the subject it poises to study, the 3T3-L1 cell model reflects the essential aspect of the biology of the adipocytes [71]. We compared the expression and binding patterns of key adipogenic transcription factors and their targets in the 3T3-L1 model to human primary adipocytes [62–64]. The expression of PPARG and CEBPA and their lipogenic gene targets LPL, ACLY and FASN was induced by the MDI in the human cells (Figure 7, *left*). In addition, PPARG had binding peaks in the promoter regions of each of the three lipogenic genes (Figure 7, *right*). The adipogenic factors also showed a pattern of binding consistent with the suggested auto-regulation and feedback loops among them [72]. These similarities support the 3T3-L1 as an *in vitro* model for studying the adipocytes.

### The curated dataset of differentiating adipocytes is publicly available and optimized for reusability

The processed RNA-Seq dataset is available as a Bioconductor experimental data package (http://bioconductor.org/packages/curatedAdipoRNA/). The package provides a SummarizedExperiment R object. The object contains three tables. The first is the metadata table, which contains the manually curated sample metadata using unified language to facilitate comparing and combining the data from different studies. The two main metadata items are the time point (hours) and the stage of differentiation of each sample (0, non-induced; 1, early; 2, intermediate; 3, late-differentiation). In addition, the metadata contain quality assessment measurements of the samples in the form of qc_read objects. The second table is a GRanges object with essential information about the genes in which reads were counted. The third table is a count matrix of all known mouse genes. Together, the tables can be used in analyses such as differential expression, gene set enrichment and/or time-course analyses.

The processed ChIP-Seq dataset is available as a Bioconductor experimental data package (http://bioconductor.org/packages/curatedAdipoChIP/). The package provides a SummarizedExperiment R object. The object contains three tables. The first is the metadata table similar to the one described above and information of the ChIP antibodies. The second table is a GRanges object with essential annotations about the Peaks in which reads were counted. The third is a count matrix of the reads in peaks and their gene assignment in the mouse genome. Together, the tables can be used in differential peak binding and occupancy analyses.

## Conclusion

We surveyed public repositories for high-throughput sequencing data on the *in vitro* adipocyte model 3T3-L1 and curated extensive metadata on the included samples. The raw data were collected and processed using standard pipelines. The product of this study is the construction of gene expression and DNA-binding models of the differentiating 3T3-L1. The processed data were documented and made available as an open-source Bioconductor experimental data packages. The models were assessed for quality and were found to reflect the essential aspect of known adipocyte biology from the published literature and the human primary adipocytes.
